# In Vivo Electroporation-Mediated, Intrahepatic Alpha1 Antitrypsin Gene Transfer Reduces Pulmonary Emphysema in Pallid Mice

**DOI:** 10.3390/pharmaceutics12090793

**Published:** 2020-08-21

**Authors:** Marco A. Sutter, Tiziana P. Cremona, Izabela Nita, Eleonora Cavarra, Giuseppe Lungarella, Eli C. Lewis, Johannes C. Schittny, Thomas Geiser, Amiq Gazdhar

**Affiliations:** 1 Department of Pulmonary Medicine, University Hospital Bern, 3010 Bern, Switzerland; marco.sutter@students.unibe.ch (M.A.S.); Izabela.Nita@dbmr.unibe.ch (I.N.); 2 Department of Biomedical Research, University of Bern, 3010 Bern, Switzerland; 3 Institute of Anatomy, University of Bern, 3010 Bern, Switzerland; tizianacremona@hotmail.com (T.P.C.);; 4 Department of Molecular and Developmental Medicine, University of Siena, 53100 Siena, Italy; eleonora.cavarra@unisi.it (E.C.); giuseppe.lungarella@unisi.it (G.L.); 5 Faculty of Health Sciences, Ben-Gurion University of the Negev, Beer Sheva 84105, Israel; eli.c.lewis@gmail.com

**Keywords:** alpha1 antitrypsin, nonviral gene delivery, electroporation, localized gene delivery, emphysema, liver gene transfer

## Abstract

Rationale: Mutation in the alpha1 antitrypsin (AAT) gene leads to low circulating levels of AAT, which is associated with several disease processes including pulmonary emphysema. The standard of care relies on substitution with plasma-purified AAT. We studied a novel approach to obtain sustained therapeutic levels of circulating AAT using nonviral in vivo electroporation-mediated gene transfer to the liver. Methods: In vivo intrahepatic electroporation-mediated human AAT gene transfer was performed in C57 Bl/6J mice carrying a genetic deficiency of murine AAT (pallid mice) and suffering from pulmonary emphysema. The animals were evaluated for lung function using flexiVent and detailed stereological assessments. Lung neutrophilic burden was assessed. Results: Pallid mice showed morphologically detectable pulmonary emphysema. Thirty days after in vivo electroporation-mediated gene transfer directly aimed at the liver, circulating human AAT was elevated and lung function was significantly improved compared to non-treated pallid mice. Stereological analysis revealed a reduction in pulmonary emphysema. Conclusion: Our data indicate that in vivo intrahepatic electroporation-mediated gene transfer of AAT is a safe and efficient procedure resulting in reduction of pulmonary emphysema in pallid mice.

## 1. Introduction

Alpha1 antitrypsin (AAT) deficiency, an autosomal codominant disease that is caused by mutation of the SERPINA1 gene, leads to liver and lung disease. The prevalence of AAT deficiency varies considerably, yet it is estimated that 3 million people carry allele combinations that are associated with severe deficiency [1]. Three major AAT variants (PI*M, PI*Z, and PI*S) are reported; of these, PI*Z is the most common and is associated with poor prognosis [2]. In AAT deficiency, there is either reduced or no secretion of AAT in circulation from liver cells. Individuals with AAT deficiency usually develop symptoms of lung involvement at older ages, which include shortness of breath, chronic bronchitis, high prevalence of bronchial obstruction with Forced expiratory volume FEV1 decline, and an ongoing decrease of computer tomography CT lung density that reflects progressive emphysematous lung destruction [3]. The dogma by which AAT deficiency is primarily a Caucasian disease has been disputed by a detailed epidemiological study [1]. Indeed, an increasing number of cases is reported for African, Asian, and Middle Eastern populations, rendering AAT deficiency a global concern. Furthermore, carriers of the alleles are at high risk of developing severe disease due to increased air pollution [4].

Current therapeutic options are comprised of lung or liver transplantation, or life-long intravenous weekly augmentation therapy with commercially available affinity-purified plasma-derived human AAT [5]. Lung or liver transplantations are associated with technical and immunological complications, and both exert high financial burden [6]. Therapeutic options that can provide sustained levels of circulating AAT for prolonged periods of time are therefore needed. In its present form, AAT augmentation therapy requires weekly intravenous administration of AAT, thus reducing quality of life. Moreover, affinity-purified AAT is a blood product and its quantities are limited on a global scale. In addition, there is the issue of nonphysiological pharmacokinetics of infused AAT: circulating levels spike for a day and then rapidly decline, exhibiting a half-life of 4.5 days followed by almost 3 days of very low levels of AAT. This is in contrast to the physiological plateau-like liver-derived circulating levels of AAT, which is apparently superior to pulse-like AAT infusions in as far as tissue protection by AAT [7]. Gene therapy offers an opportunity to provide sustained levels of AAT, with possible therapeutic benefit and greater patient compliance.

Clinical trials for using gene therapy in AAT deficiency have been running for longer than the past decade since the condition is a well-recognized one-gene disease. Two trials used Adeno-Associated Virus (AAV), while the third trial used nonviral cationic liposomes [7]. AAV was injected intramuscularly, while liposomes were administered as droplets in the nostrils. Both of these vectors are limited due to dose-dependent toxicity and low transgene expression levels and rely on nonphysiological extrahepatic production of AAT. Thus, a more relevant and feasible approach for achieving gene transfer is required.

In vivo electroporation-mediated gene transfer is based on the application of short electric pulses. This method has been shown to be effective in various disease models [8,9,10,11] and has recently been applied in clinical practice [12]. While the main source of native AAT is the liver, most other studies have focused on gene delivery for systemic distribution (e.g., muscle tissue) or in a lung-directed manner [7].

In the current study, the outcomes of electroporation-mediated AAT gene therapy in the liver is tested in mice that carry a mutation in the pallid gene. The pallid mutation hinders normal secretion of AAT in circulation, leading to spontaneous development of pulmonary emphysema [13]. We thus evaluated clinically relevant outcomes of in vivo intrahepatic electroporation-mediated AAT gene delivery in this model.

## 2. Materials and Methods

### 2.1. Plasmid

Plasmids pEF-AAT carrying the complete human SERPINA1 into pEF (plasmid with eukaryotic translation elongation factor 1 alpha 1 promotor region), containing the Epstein barr virus sequence and human gene (SERPINA1) encoding1-antitrypsin (AAT) as described by Stoll SM et al. [14], was used for this study. The plasmid consists of full SERPINA1 on a 19-kb genomic fragment and the Epstein Barr Virus (EBV) gene EBNA1. For large-scale plasmid production, we used the Giga prep kit (Qiagen, Germantown, MD, USA) following manufacturer’s protocol. For electroporation, the plasmid was diluted in distilled water to a concentration of 1 µg/µL.

### 2.2. Pallid Mice

C57BL/6J-backcrossed pallid mice (pa/pa) were obtained from the laboratory of G Lungarella (University of Sienna, Sienna, Italy). The mice were housed in an environment controlled for light (7 am to 7 pm) and temperature (18 °C to 22 °C); food (Mucedola Global Diet 2018; Harlan, Corezzana, Italy) and water were provided for consumption ad libitum. Experiments were performed in accordance with the standards of the European Convention of Animal Care. The study protocol was approved by the Animal ethics committee Canton Bern and by the University of Bern Animal Study Committee (BE 56/15, date: 17 May 2016).

Pallid mice (10–12 months old, female) were studied. Animals were randomly divided into three groups: (a) no treatment (Pa/Pa), (b) empty vector electroporation (empty vector), and (c) pEF-AAT plasmid (AAT plasmid) electroporation, n = 5 in each group. Electroporation-mediated pEF-AAT or empty vector was performed twice at days 0 and 15 after the first procedure. Animals were sacrificed after day 30 from the first electroporation. Age- and gender-matched wildtype C57BL/6J mice served as controls (wildtype, WT) (n = 5).

## 3. Electroporation-Mediated Gene Transfer to the Liver

Mice were injected with buprenorphine (0.1 mg/kg) subcutaneously 30 min before surgery. For anaesthesia, mice received midazolam (Dormicum) 5 mg/kg, medetomidine (Domitor) 0.5 mg/kg, and fentanyl (Fentanyl-Janssen) 0.05 mg/kg intraperitoneally. For eye protection, bepanthen (5% Dexpanthenol) was applied to the eyes. For the electroporation procedure, sterile conditions were applied. The fur from the abdominal area was shaved, and the skin was disinfected using betadine (Mundipharma, Basel, Switzerland). A midline incision was performed, and the liver was mobilized using cotton sticks. The caudate lobe was pulled, and 50 µL of plasmid (empty vector or pEF-AAT (1 µg/µL)) was injected using an insulin syringe (BD Ultrafine 6 mm, BD biosciences, San Jose, CA, USA). Immediately thereafter, the tweezer electrode (1 cm × 1 cm) was placed directly over the injected area and electroporation was performed using super electroporator NEPA 21 type II (NEPAGENE Ltd., Chiba, Japan) ×8 pulses of 200 v/cm of 10 millisecond duration at intervals of 50 milliseconds of poring pulses and ×8 pulses of 200 v/cm of 50 millisecond duration at 50 millisecond intervals of transfer pulses, following the optimized parameters as described before [15]. After electroporation, the liver was carefully pushed back into place using cotton sticks and the abdomen was stitched closed by two layers using absorbable 4-0 silk suture (Ethicon, Cincinnati, OH, USA). Mice were allowed to recover while receiving intraperitoneal atipamazol (Revetor) 2.5 mg/kg, naloxon (Narconti) 1.2 mg/kg, and flumazenil (Anexata) 0.5 mg/kg. Buprenorphine (0.1 mg/kg) was administered for 3 days after surgery. An appropriate score sheet was maintained to monitor the well-being of the mice.

## 4. Assessment

### 4.1. Lung Function

Mice were anaesthetized with a mixture of midazolam (5 mg/g), fentanyl (50 µg/g), and medetomidin (0.5 µg/g), were intratracheally intubated, and were connected to a computer-controlled small animal ventilator (flexiVent, SCIREQ, Montreal, Canada) at a frequency of 120 breaths/min and tidal volume of 10 mL/kg. After stabilization, lung mechanics were measured at positive end-expiratory pressure (PEEP) levels of 3 cm H_2_O. A pressure volume (PV-P) perturbation was performed to collect lung mechanic data, i.e., static compliance; A- and K-factor of the Salazar–Knowles equation; and area, i.e., the amplitude of the hysteresis prior to PV loop.

### 4.2. Tissue Collection

After lung function assessments, the animal was sacrificed. Bronchioalveolar lavage, blood, and lung and liver tissue were collected for further analysis.

### 4.3. Histology and Microscopy

Paraffin-embedded lung sections were stained with haematoxylin and eosin (H&E). For stereological analysis, images were acquired using ColorView IIIu digital camera (Olympus Soft Imaging Solutions, Münster, Germany) on a Leica DM RB light microscope (Leica, Wetzlar, Germany) equipped with an automated motorized stage at magnification of ×20. For overviews, the images were acquired by Pannoromic 250 II flash scanner (3D HISTECH Ltd., Budapest, Hungary) at ×20 magnifications. Immunofluorescence images were acquired using LSM 710 confocal microscope (Zeiss, Ulm, Germany).

### 4.4. Stereological Analysis

Stereological measurements were performed in compliance with the standards for quantitative assessment of the American Thoracic Society (ATS) and European Respiratory Society ERS [16]. Immediately after lung function evaluation, the animal was sacrificed and the left lung was prepared for stereological analysis. The left lobe was inflated with paraformaldehyde 4% at a pressure equivalent to 20 cm H_2_O; when completely expanded, the trachea was ligated and stored in 4% paraformaldehyde for histological experiments. Left lung volume was determined by the water-displacement method [17]; fixed lungs were embedded in 2.5% agar and cut in transverse slices of 1.2 mm. About 6–9 pieces were obtained and paraffin-embedded; 5-µm sections were stained with H&E. One hundred micrographs were captured for each animal using a ColorView IIIu digital camera (Olympus Soft Imaging Solutions) on a Leica DM RB light microscope equipped with an automated motorized stage at a final magnification of ×20. Images were quantitatively analysed using a test system of points and lines superimposed over the digital images via the STEPanizer (software designed by Stefan Tscahnz, University of Bern, Bern, Switzerland) [18]. Point counts yielded relative volume densities and were categorized as outside, artefacts, septa, infiltrated septa, or nonparenchyma. Parenchymal air space proportion was calculated by subtracting points falling on septa from all points hitting the parenchyma. Absolute volumes of air spaces and septa were computed by multiplying volume densities by absolute lung volume [19,20]. Mean linear intercept and septal surface area were calculated using line counts intersecting septa and by multiplying by lung volume. The right lobes of the lung were ligated at the right bronchus and snap-frozen for RNA/protein extraction in Tissue teck OCT (Optimal cutting temperature compound) (Shakura Finetek, Alphen aan den Rijn, Netherlands).

### 4.5. Sirius Red Staining

Liver tissue was cryopreserved using Tissue-tec OCT compound (Genprice Inc. San Jose, CA, USA), and 10-µm cryosections were stained with Sirius Red staining (Sigma Aldrich, USA) to evaluate the effect of electroporation on liver collagen according to the manufacturer’s instructions.

### 4.6. Immunofluorescence Staining

Cryosections were fixed with 70% ethanol, washed with 1 × PBS thrice, and incubated with primary antibody recombinant anti-alpha1 antitrypsin antibody (Abcam, Cambridge, UK) at a dilution of 1:100 overnight at 4 °C. After the 1 × PBS wash, the slides were treated with Fluorescein isothiocyanate (FITC) (anti-rabbit) secondary antibody at a dilution of 1:1000 and were incubated in a moist chamber for 2 h at room temperature; the slides were washed with 1 × PBS and mounted using the glycerol mounting media-anti fade with (4′,6-diamidino-2-phenylindole) DAPI (Abcam, Cambridge, UK). Zeiss LSM 710 (Karl Zeiss, Ulm, Germany) confocal microscope was used for visualization.

### 4.7. Liver Homogenization

Liver was resected and frozen in liquid nitrogen; 100 mg of frozen liver was homogenized in 100 uL protein lysis buffer (Pierce Lysis buffer, Thermo-Fischer Scientific, Waltham, MA, USA) and homogenized using polytron for 20 s. The homogenate was centrifuged at 12,000 rpm for 20 min to remove debris. The cleared lysate was used for further analysis. Total protein was measured using the standard Bradford method (BioRad, Irvine, California, USA).

### 4.8. ELISA

Human AAT was measured in the serum and in the liver homogenate of mice using Human A1AT ELISA kit (ICLLAB, Portland, OR, USA), and mice AAT in the liver homogenate of mice was measured using the mouse alpha1 antitrypsin ELISA kit (Abcam, Cambridge, UK) following the protocol provided.

### 4.9. Myeloperoxidase Activity

Activity of neutrophil-specific myeloperoxidase enzyme (MPO) was measured by an adaptation of the method described by Bradley et al. [21] and modified by Mullane et al. [22]. MPO activity was measured in bronchioalveolar lavage BAL fluid. The sample was mixed with o-dianisidine, and the reaction was started by adding H_2_O_2_. The reaction was then stopped by addition of NaN_3_ solution after 3 min of incubation, and optical densities (ODs) were measured at 450 nm. To quantify MPO activity, a standard curve was obtained. MPO values are expressed as mg/mL.

### 4.10. Measurement of Liver Injury

Serum samples were collected to assess the extent of liver injury after electroporation-mediated gene transfer using enzymatic assays to measure albumin, ALT (alanine aminotransferase), and AST (aspartate aminotransferase) (P800; Modular Analytics EVO, Roche, Mannheim, Germany).

## 5. Statistics

Data are presented as mean ± SD. We used one-way ANOVA and multiple comparison test or unpaired t test using GraphPad Prism 7 (GraphPad Software, San Diego, CA, USA). Results were considered significant at *p* < 0.05.

## 6. Results

Human AAT is detected in the liver and sera of mice 30 days after intrahepatic electroporation-mediated AAT gene transfer.

**Figure 1:**
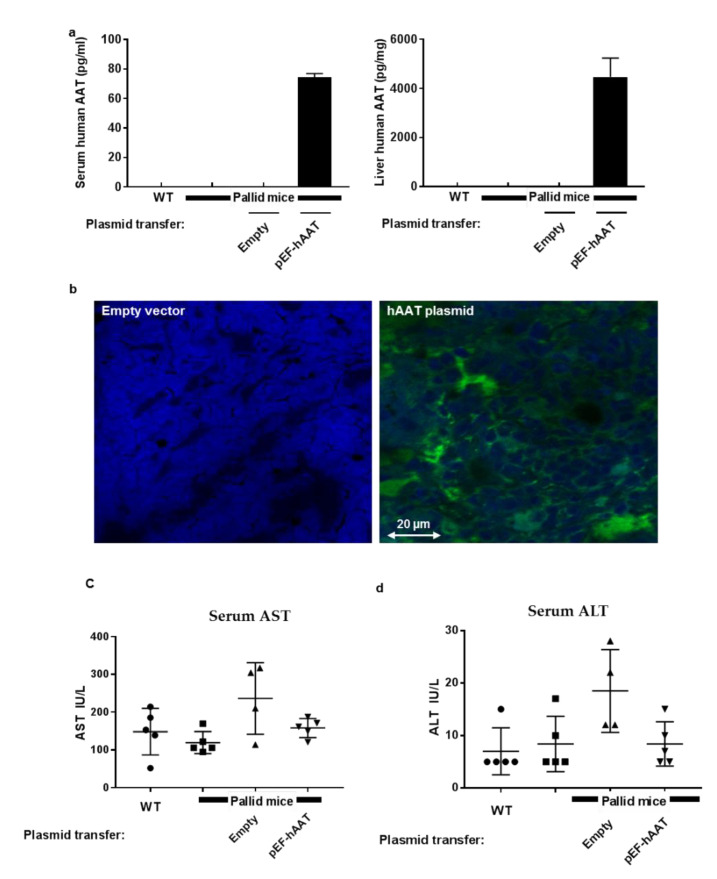

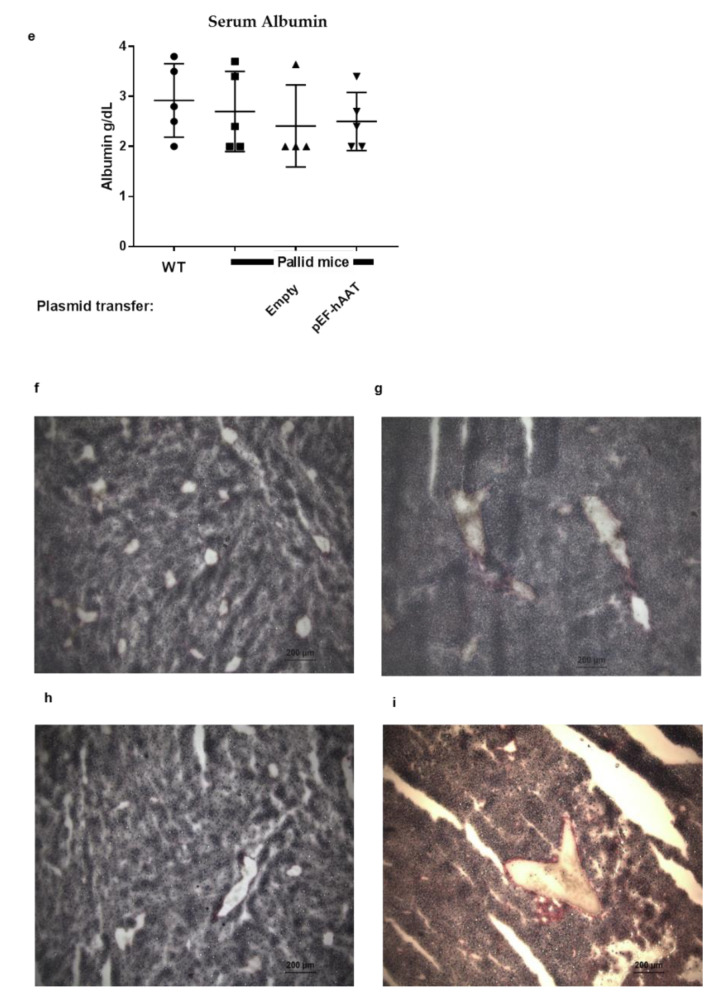
Human alpha1 antitrypsin (AAT) expression 30 days after in vivo intrahepatic electroporation-mediated hAAT gene transfer: (**a**) hAAT levels in serum and liver homogenates, mean ± SD; (**b**) representative immunofluorescent images of the liver 30 days after intrahepatic electroporation-mediated gene transfer, green fluorescence: hAAT. Electroporation-mediated gene transfer is safe in the liver. Serum levels of liver enzymes were measured: (**c**) serum aspartate aminotransferase (AST), (**d**) serum alanine aminotransferase (ALT), and (**e**) serum albumin. Values are expressed as mean and ± SD; all the values are within physiological range. Sirius red staining of the liver section did not show any sign of liver injury or fibrosis after electroporation-mediated gene transfer as observed by Ishak system staining [23]. Wildtype (**f**), pallid (**g**), empty vector (**h**), and pEF-hAAT (**i**).

In order to characterize the production of human AAT after electroporation-mediated DNA delivery to the liver, pallid mice underwent the procedure with either an empty vector or a pEF-hAAT plasmid; for controls, wildtype mice and pallid mice were included in data collection (n = 5 per group). As shown (Figure 1), human AAT production was readily detected 30 days after human AAT gene transfer, as determined in liver homogenates (4463 ± 1356 pg/mg protein) and sera (75 ± 3.4 pg/mL) (Figure 1a). Human AAT was undetectable in the control groups. Accordingly, immunofluorescent staining depicted the expression of hAAT by mice liver cells with the anticipated cytoplasmatic distribution of the protein (Figure 1b). Interestingly, mice AAT levels measured in liver did not show any change among the different groups (data shown in the Supplementary Materials (Figure S1)).

### 6.1. Lung Function Is Improved after Intrahepatic Electroporation-Mediated AAT Gene Transfer

**Figure 2:**
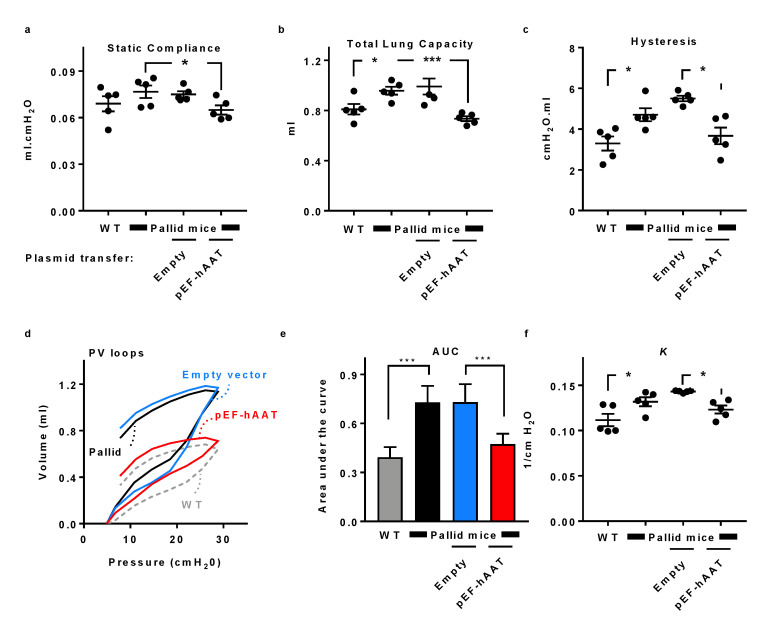
Lung function in pallid mice after intrahepatic electroporation-mediated hAAT gene transfer. Respiratory parameters were performed on a flexiVent system (SCIREQ): static compliance, total lung capacity, and hysteresis were obtained in tracheotomize mice. B6, C57BL/6 mice; Pa, pallid mice; EV, pallid mice treated with an empty vector; AAT, pallid mice treated with AAT plasmid (*n  = * 5 per group). Lung function measurements: (**a**) static compliance, (**b**) total lung capacity, and (**c**) hysteresis (area under the (pressure volume) PV loop). Static compliance was calculated from the deflating part of the PV loop between 3–7 cm H_2_O, total lung capacity was measured using a deep inflation manoeuvre, and hysteresis represents the area between the inflating and deflating parts of the PV loop. Each symbol represents the average of three correct manoeuvres per mouse. Values are shown as mean ± SD; * *p* < 0.05, *** *p <* 0.001 compared to untreated control group. (**d**) Pressure-volume (PV) loops, (**e**) analysis of the PV-loops, and (**f**) parameter *K* representing the curvature of the PV loops curves: Two-way ANOVA applying Bonferroni’s multiple comparisons test compared the mean inspiratory volume of four groups (used at each pressure step up to 30 cm H_2_O). Statistical significance * *p* < 0.05. t-tests were used in B and C. Values are expressed as mean and ± SD * *p* < 0.05, *** *p <* 0.001 compared to the untreated control group.

In order to examine whether AAT gene transfer has an impact on lung function, we measured basal lung functions in mice (Figure 2). High lung tissue compliance indicates a lung with low elastic recoil, typical in mice that develop emphysema. As shown (Figure 2a), static compliance (Cst) was higher in pallid mice compared to wildtype (C57BL/6J) mice as well as to pallid mice transfected with an empty vector. However, 30 days after AAT gene transfer to pallid mice, Cst was reduced to the level of wildtype mice. Similarly, compliance under mechanical stress (Figure 2b), a measurement of total lung capacity (i.e., A-factor), was higher in pallid mice compared to wildtype mice, agreeing with the presence of pulmonary emphysema. Pallid mice displayed a significant reduction in lung capacity after AAT gene transfer, depicting values in the range of healthy wildtype mice. Estimating atelectasis that exists before the PV loop, hysteresis was higher in pallid mice compared to wildtype mice; however, after AAT gene transfer, it was reduced towards the range observed in healthy wildtype mice (Figure 2c). Consistent with these results, pallid mice exhibited an upward and leftward shift in PV relation compared to wildtype mice and compared to mice receiving AAT gene transfer; significant differences were found in the area between the inflation and deflation limb of the PV curves (Figure 2d,e) and in the shape constant k that describes the curvature of the PV loop curves (Figure 2f). This indicates that treatment with AAT gene therapy changed the intrinsic elastic properties of the respiratory system. The values are shown in Table 1.

### 6.2. Stereological Analysis Reveals Improved Lung Architecture after AAT Gene Transfer

**Figure 3:**
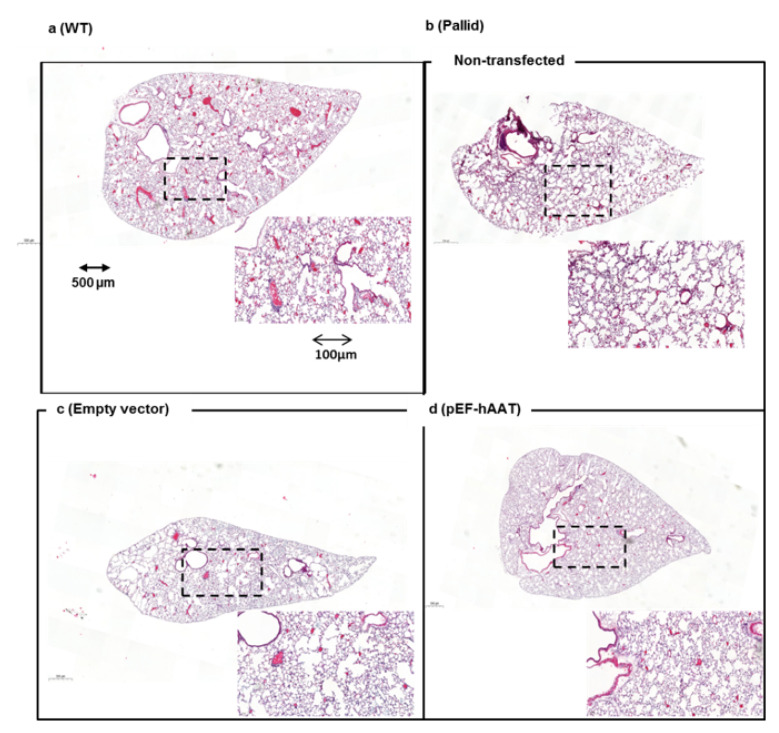
Left lobe lung histology after intrahepatic AAT gene transfer by electroporation in vivo: Representative micrographs of the lungs of animals from different groups. (**a**) Normal lung from wildtype (WT) (C57BL/6J) mice, (**b**) air space enlargement visible in the non-transfected pallid mice and (**c**) in the animals treated with empty vector, (**d**) animals treated with the pEF-AAT plasmid: ×20 magnification, scale bar 500 µm. Inset, images at higher magnification as indicated by dashed frame (digital zoom; scale bar 200 µm).

**Figure 4:**
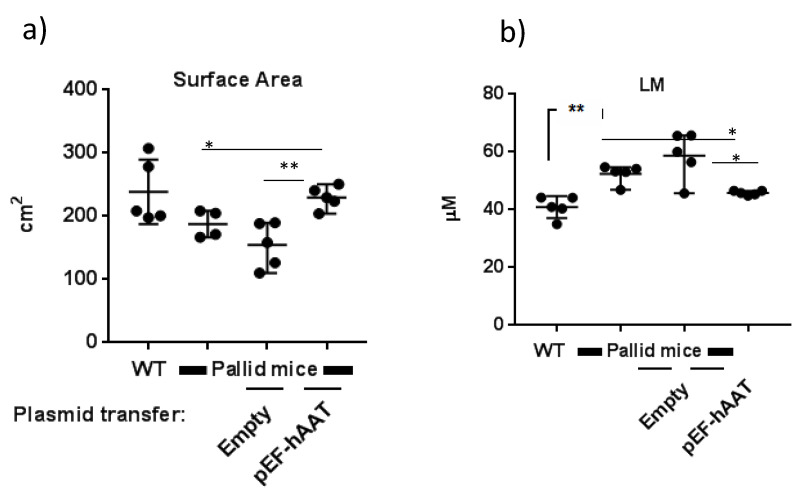
Lung morphometry: (**a**) septal surface area and (**b**) mean linear intercept (LM) in different experimental groups, as indicated. For each animal, the left lobe was analysed, and all parameters relate to this specific lobe. Statistical values are expressed as mean ± SD, unpaired t test was performed, and each group was compared to each other. * *p* < 0.05, ** *p <* 0.01.

Histological evaluation of the lungs of pallid mice at 12 months of age revealed the presence of emphysema, characterized by an enlargement of the distal airspaces and loss of the normal alveolar structures as compared with wildtype mice (Figure 3a,b). An evident reduction of alveolar space enlargement was observed in the distal zones of the lung of pallid mice after AAT gene transfer as compared to mice treated with an empty vector (Figure 3c,d). Surface area density and mean linear intercept LM values of pallid mice at 12 months reflect a loss of alveolar tissue in the lung parenchyma. These changes are not evident in mice treated with AAT gene therapy compared to the mice treated with control plasmid (Figure 4a,b). Values are shown in Table 2. Additional data is presented in the Supplementary Materials as Figure S1 and Table S1.

### 6.3. AAT Gene Transfer by Electroporation Did Not Induce Liver Injury

The effect of electroporation on liver function was assessed by measuring serum AST (Figure 1c), ASL (Figure 1d), and albumin (Figure 1e); all values were within the normal range as shown before [24]. Moreover, Liver cryosections were stained for evaluation of collagen by Sirius red staining and observed under the microscope for Ishak system staining [23]; no signs of fibrosis were detected in any group (Figure 1f–i).

### 6.4. Lung Neutrophil Burden Is Reduced after AAT Gene Transfer

**Figure 5:**
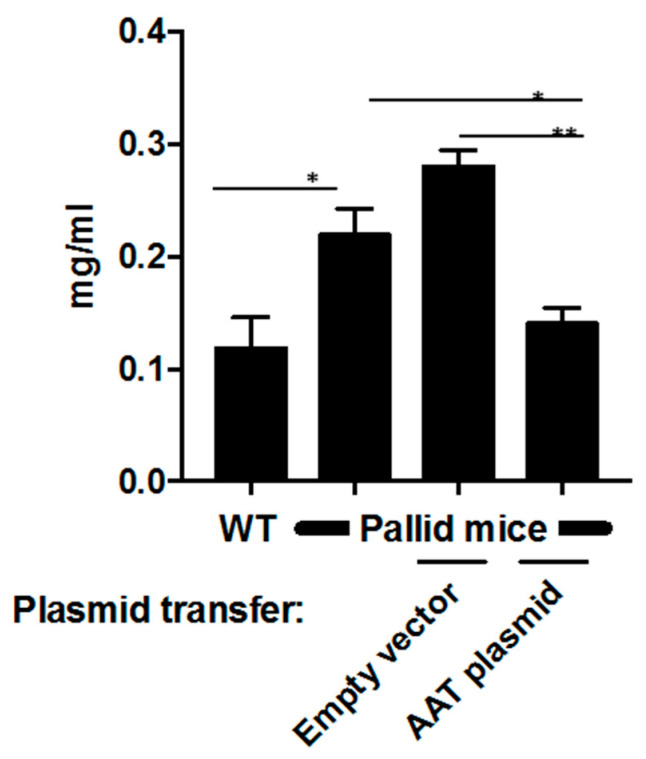
Bronchioalveolar lavage (BAL) fluid neutrophilic activity: After electroporation-mediated hAAT gene transfer, myeloperoxidase assay (MPO) was performed to assess the neutrophil activity in BAL fluid. Data are shown as mean ± SD, unpaired t test was performed, and each group was compared to each other * *p* < 0.05, ** *p* < 0.01.

Neutrophil activity was depicted by measuring MPO levels (Figure 5). The baseline MPO in wildtype mice was 0.114 ± 0.032 mg/mL. However, MPO activity in pallid mice was 0.220 ± 0.050 mg/mL and that in animals treated with an empty vector was 0.287 ± 0.085 mg/mL; in contrast, after AAT gene transfer, MPO levels were significantly reduced to 0.147 ± 0.040 mg/mL (*p* < 0.05).

## 7. Discussion

The present study provides evidence that in vivo intrahepatic electroporation-mediated AAT gene transfer improves lung function and morphology and reduces neutrophil activity in a mouse strain that models human AAT deficiency, i.e., pallid mice.

Gene therapy has been tested for AAT deficiency in preclinical studies using various animal models and using either viral vectors [25,26,27,28,29] or nonviral vectors, e.g., liposomes [30,31,32]. For clinical trials, however, different serotypes of AAV have been used [33,34] by either intramuscular or intrapleural routes. Aside from the disadvantages of viral interventions or intrapleural delivery profiles, AAT is primarily synthesized by hepatocytes, which hold inherent and optimal processing steps for secretion of mature AAT. Circulating AAT provides systemic tissue protection, predominantly in the form of a superior balance between protease and antiprotease activities as well as protection of lung tissue from excessive neutrophil elastase activity. Therefore, targeting the liver for gene transfer would be a more physiological choice.

Gene therapy that is aimed at chronic diseases would benefit from persistent gene expression, requiring a promoter that is preferably cell specific and holds extrachromosomal replication mechanisms for prolonged expression. In the current study, we introduced a human hepatocyte-directed pEF-AAT plasmid directly to the liver by applying in vivo electroporation-mediated gene transfer. The animals survived the procedure with no complications, asserting the safety of in vivo electroporation for the liver [15,35] and possibly other organs [8,9,10,11,36]. The issue of organ distribution of the plasmid deserves further exploration, with the goal of developing a method whereby a larger area of liver can be introduced with a plasmid.

In a recent phase 2a clinical trial, AAV was injected intramuscularly and resulted in elevated levels of circulating AAT in 3 out of 9 patients for a period of 5 years [37]. Despite these promising results, it is not clear what led to increased tolerogenic responses in the positive producers. Nonetheless, the advantage of a nonviral electroporation method is the lack of an immune provocation which should therefore be tested in the clinical setup in the future.

In the current study, we demonstrate improved lung function in animals receiving intrahepatic electroporation-mediated AAT gene transfer. In untreated animals, reduced static compliance parameter showing lung expandability, reduced inspiratory capacity, and reduced hysteresis as a measure of atelectasis were observed. Compared to these findings, the PV loop showed a downward shift in the animals receiving AAT gene transfer. Also, the lung architecture showed a trend towards improvement, and most importantly, no further structural deterioration was observed after electroporation-mediated AAT gene transfer.

The current study is not without limitations. The procedure of in vivo electroporation is invasive, and minimally invasive methods need to be applied that can target the liver. One possibility might be the application of electrospray [38], a method that relies on the principle of columb repulsion and can be delivered using single port device. In addition, the experimental endpoint was collected one month after the first treatment; a long-term study is warranted to see the prolonged effect of this approach. Also, at this proof-of-concept stage of study, we did not fully explore options for increasing the levels of human AAT in circulation. The relatively low levels of circulating human AAT under the presently studied settings may be the result of several limitations: (1) the mice could generate antihuman antibodies against human AAT; (2) the pallid mutation could interfere with AAT secretion; (3) dynamics of plasmid expression might reveal suboptimal production of human AAT on day 30 as per the present report, and (4) volumes and doses of plasmid administration should be further explored. Nonetheless, the fact that the outcome of the described gene delivery method resulted in an altered profile of emphysema suggests that the levels measured on day 30 might be sufficient for achieving a biologically relevant impact. Moreover, to further evaluate the effect of AAT gene transfer on lung architecture, a long-term study will be needed specially as a prophylaxis and a gene transfer procedure before emphysema starts to develop in pallid mice [39] should be performed.

## 8. Conclusions

In conclusion, intrahepatic electroporation-mediated AAT gene transfer is a feasible, safe, and reproducible approach in the context of a mouse model of AAT deficiency with spontaneous pulmonary emphysema. In addition to improved lung function, electroporation-mediated AAT gene transfer effectively reduces local neutrophil activity. While the current study offers a good model for correction of lung damage in AAT deficiency, a more elaborate preclinical study is required using large animal models before clinical translation is considered.

## Figures and Tables

**Table 1:** Parameters for the lung function test (Figure 2): data are presented as mean ± SD.

Parameters	WT (Wildtype)	Pallid Mice	Empty Vector	hAAT Plasmid
Static Compliance (mL/cmH_2_O)	0.068 ± 0.004	0.0767 ± 0.004	0.0749 ± 0.001	0.064 ± 0.002
Total Lung capacity (A) (mL)	0.80 ± 0.04	0.95 ± 0.03	0.99 ± 0.06	0.73 ± 0.01
Hysteresis (cmH_2_O/mL)	3.29 ± 0.34	4.70 ± 0.31	5.50 ± 0.13	3.66 ± 0.40
AUC (area under curve) (mL/cmH_2_O)	0.39 ± 0.06	0.72 ± 0.10	0.73 ± 0.11	0.47 ± 0.06
k (curvature of the upper portion of the deflation limb of the pressure volume (PV) curve)	0.11 ± 0.006	0.13 ± 0.004	0.143 ± 0.0005	0.12 ± 0.004

**Table 2:** Parameters for stereology (Figure 4): data are presented as mean ± SEM.

Parameters	WT	Pallid Mice	Empty Vector	hAAT Plasmid
Surface Area (cm^2^)	238.2 ± 22.76	187.6 ± 8.43	154.4 ± 16.09	229.6 ± 7.94
Mean liner intercept (LM) (µM)	40.9 ± 1.68	52.38 ± 1.42	58.68 ± 3.69	45.79 ± 0.33

## Supplementary Materials

The following are available online at https://www.mdpi.com/1999-4923/12/9/793/s1, Figure S1: Lung morphometry: Stereological analysis of fluid displacement, total volume parenchyma, total septal volume and serum level of mice AAT, Table S1: Stereology additional data.
