# Supplementary material for: In Vivo Electroporation-Mediated, Intrahepatic Alpha1 Antitrypsin Gene Transfer Reduces Pulmonary Emphysema in Pallid Mice
Source: Pharmaceutics. 2020 Aug 21;12(9):793. doi: 10.3390/pharmaceutics12090793 (PMC7559762; doi:10.3390/pharmaceutics12090793)
Supplement: Supplementary file 1 [file pharmaceutics-12-00793-s001.pdf]

pharmaceutics

MDPI

In Vivo Electroporation-Mediated, Intrahepatic Alpha1 Antitrypsin Gene Transfer Reduces Pulmonary Emphysema in Pallid Mice

Marco A. Sutter, Tiziana P. Cremona, Izabela Nita, Eleonora Cavarra, Giuseppe Lungarella, Eli C. Lewis, Johannes C. Schittny, Thomas Geiser and Amiq Gazdhar

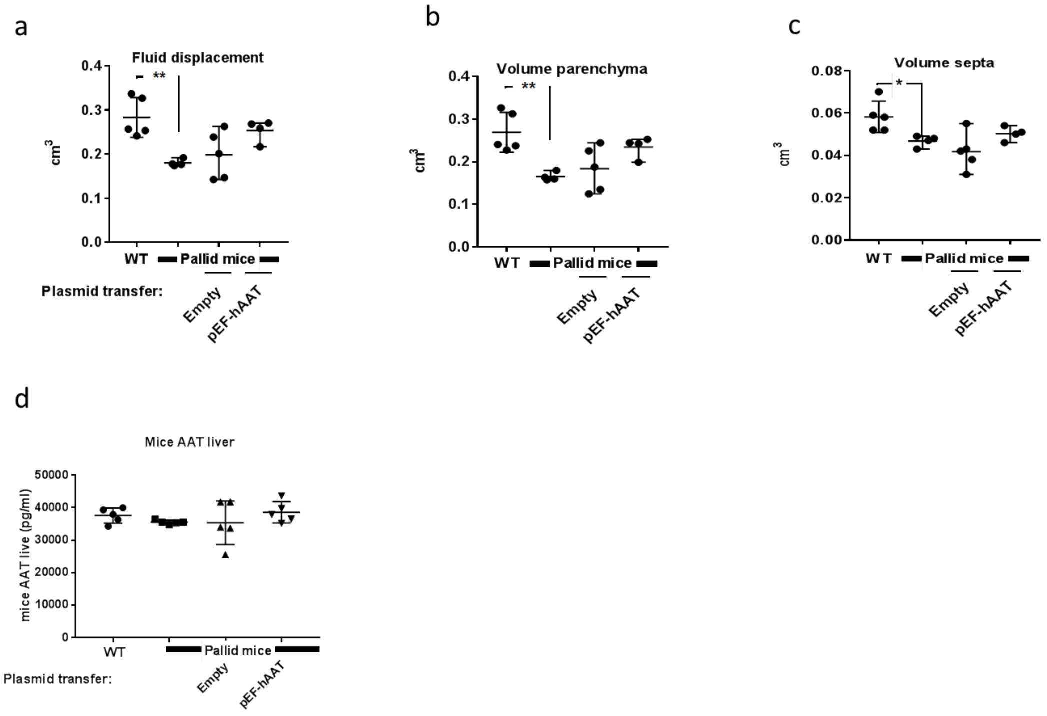

Figure S1. Lung morphometry: Stereological analysis of (**a**) fluid displacement, (**b**) total volume parenchyma, (**c**) total septal volume and (**d**) serum level of mice AAT. * $p < 0.05$, ** $p < 0.01$.

Table S1. Stereology additional data.

Parameters	WT (wildtype)	Pallid mice	Empty vector	hAAT plasmid
Fluid displacement (cm^3)	0.28 ± 0.02	0.18 ± 0.004	0.19 ± 0.02	0.25 ± 0.01
Volume Parenchyma (cm^3)	0.26 ± 0.02	0.16 ± 0.004	0.18 ± 0.02	0.23 ± 0.01
Volume Septa (cm^3)	0.05 ± 0.003	0.04 ± 0.001	0.04 ± 0.003	0.05 ± 0.001
